# Global and Partial Effect Assessment in Metabolic Syndrome Explored by Metabolomics

**DOI:** 10.3390/metabo13030373

**Published:** 2023-03-02

**Authors:** Marion Brandolini-Bunlon, Benoit Jaillais, Véronique Cariou, Blandine Comte, Estelle Pujos-Guillot, Evelyne Vigneau

**Affiliations:** 1 Université Clermont Auvergne, INRAE, UNH, Plateforme d’Exploration du Métabolisme, MetaboHUB Clermont, 63000 Clermont-Ferrand, France; 2 Oniris, INRAE, StatSC, 44300 Nantes, France

**Keywords:** multiblock, metabolomics, clinical data, global effect, partial effect, partial correlation, metabolic syndrome, NuAge cohort

## Abstract

In nutrition and health research, untargeted metabolomics is actually analyzed simultaneously with clinical data to improve prediction and better understand pathological status. This can be modeled using a multiblock supervised model with several input data blocks (metabolomics, clinical data) being potential predictors of the outcome to be explained. Alternatively, this configuration can be represented with a path diagram where the input blocks are each connected by links directed to the outcome—as in multiblock supervised modeling—and are also related to each other, thus allowing one to account for block effects. On the basis of a path model, we show herein how to estimate the effect of an input block, either on its own or conditionally to other(s), on the output response, respectively called “global” and “partial” effects, by percentages of explained variance in dedicated PLS regression models. These effects have been computed in two different path diagrams in a case study relative to metabolic syndrome, involving metabolomics and clinical data from an older men′s cohort (NuAge). From the two effects associated with each path, the results highlighted the complementary information provided by metabolomics to clinical data and, reciprocally, in the metabolic syndrome exploration.

## 1. Introduction

In precision medicine, the ultimate goal is to decipher disease phenotypes in order to improve diagnosis and treatment. Advances in deep phenotyping approaches, in particular using -omics technologies, allowed the emergence of systems biology as an integrated perspective to achieve more precise modeling of complex diseases [1]. Clinical syndromes are defined as a group of signs and symptoms that occur together and characterize a particular biological abnormality (https://disease-ontology.org/, accessed on 31 January 2023). From a numerical point of view, they are defined by a cluster of quantitative clinical variables with specific cut-offs defining the binary outcome. Untargeted metabolomics is now routinely used as a powerful tool to get an integrated view of biological systems, better understand complex phenotypes, discover biomarkers and validate patterns that are characteristic of particular biological states in various populations. However, it generates high-dimensional data that need dedicated treatment to extract biological knowledge. The common strategy for processing such data consists in performing univariate and multivariate statistics to reveal variables of interest that will be further used for biological interpretation [2]. Moreover, in health-related case-control studies, untargeted metabolomics is often integrated with standard clinical information in order to better predict and understand clinical syndromes or diseases of interest. However, extracting correlations as meaningful biological interactions is not trivial, and deciphering the modulation of metabolites from clinical factors is of major importance to achieve more precise modeling of clinical syndromes [3].

From a data analysis point of view, this leads to setting up a supervised model with two blocks of input data (metabolomics, clinical characteristics) being potential predictors of the targeted output to be explained. Such a configuration can be represented with a path diagram where directional links connect each input block to the output. In health-related studies, metabolomics reflects the clinical state to a certain extent suggesting that the input blocks are also interrelated. In this a priori-drawn path diagram, we were interested in evaluating the effect of an input block, either on its own (global effect) or conditionally to another (partial effect), on the output response. In the classical path analysis approach that could be used for such multiblock modeling, the data blocks must be unidimensional, and ‘direct’, ‘indirect’ and ‘total’ effects are estimated on the basis of standardized coefficients (path coefficients) in linear regression models [4,5]. Indeed, the regression coefficient approach is not suitable when the explanatory variables are highly colinear. Due to the multidimensionality of the metabolomic data, the use of PLS regression models is advocated to evaluate the links between the data blocks. Global and partial effects are then defined from the explained variance accounted for each model.

In the present work, published data from a project on metabolic syndrome (MetS) within the NuAge longitudinal cohort on aging [6] were used as a case study [7]. In this publication by Comte et al. [7], data were acquired by different untargeted metabolomic methods combined in a multiplatform approach followed by a variable selection strategy to build a comprehensive molecular signature of the metabolic syndrome, including 102 metabolites. The objective of our study was to enrich our knowledge about MetS by the assessment and explanation of global and partial effects in path diagrams involving the same metabolomics and clinical input blocks and the same output response consisting of a binary variable indicating the MetS presence (case or control). Then, we sought to identify the most important variables in the global effect and study the effect of introducing the mediating block. As in this study, there were no obvious causal and/or temporal links between clinical and metabolomic perturbations, we adopted a data-driven approach, and two pathway diagrams were therefore studied, differing in the mediating block, which was either the metabolomics or the clinical data.

## 2. Materials and Methods

### 2.1. Experimental Design

#### 2.1.1. Available Data

Published data from a project on MetS within the Quebec Longitudinal Study on Nutrition and Successful Aging (NuAge) [6]; (https://nuage.recherche.usherbrooke.ca/en/, accessed on 31 January 2023) were used as a case study, including 121 male subjects [7]; 2 subjects were removed after participant’s withdrawal. A binary variable, **y**, indicated the subjects’ status regarding the MetS presence (case or control).

A clinical data block (**Clinic**) included the 6 quantitative MetS diagnostic variables collected at baseline, scaled to unit variance. In the present work, only subjects with no missing values (54 cases/45 controls) were kept for analysis.

A metabolomic data block (**Metabo**) included a comprehensive MetS signature of 102 selected variables from serum sample analyses at baseline. The data acquisition, processing, and feature selection strategy, as well as annotations of these 102 variables, are provided in Comte et al. (2021) [7]. In the present work, null intensities within the metabolomic dataset were replaced by 80% of the minimum intensity value of the corresponding variable before a logarithm transformation.

#### 2.1.2. Path Diagrams

**Figure 1:**
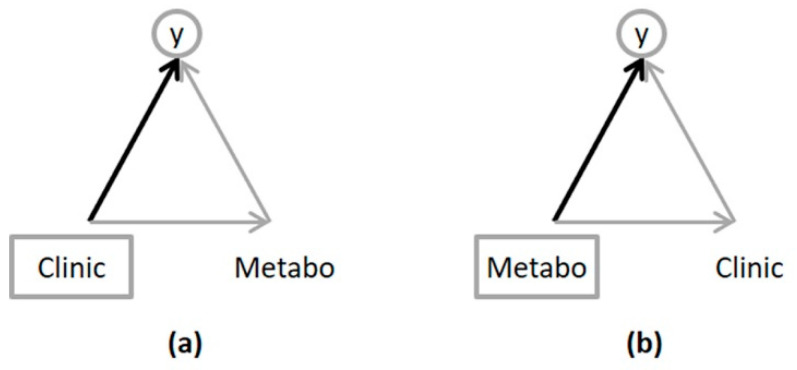
Path diagrams: (**a**) path 1; (**b**) path 2. The output variable is circled, and the input block is in a rectangle.

The “global” and “partial” effects of an input block on an output response were computed by considering two different path diagrams, named “path 1” and “path 2”, respectively (Figure 1). The output response, **y**, was a binary variable indicating the MetS presence. The two explanatory, or input, data blocks were metabolomics and clinical datasets, named **Metabo** and **Clinic**, respectively. In path 1, **Clinic** predicts **y** with **Metabo** as a mediating block. In path 2, **Metabo** predicts **y** with **Clinic** as a mediating block.

### 2.2. Effect Assessment

#### 2.2.1. Effect Calculation

**Figure 2:**
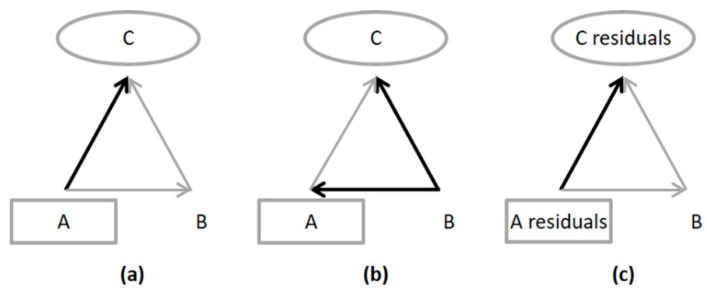
Regression models: (**a**) in the global effect calculation; (**b**) in the first step of the partial effect calculation; (**c**) in the second step of the partial effect calculation.

In the framework of multiblock analysis, directed acyclic graphs (DAG) are convenient ways to represent the conditional dependence relations between blocks. Let us consider a graph with three vertices representing three different data blocks, denoted A, B and C (as in Figure 2a,b). Furthermore, suppose three directed arrows between these vertices indicate a direct dependence from A to C and an indirect link connecting A to C through B.

With respect to the DAG depicted in Figure 2a, A refers to an independent block (that is to say, an explanatory one), C a dependent block (i.e., to be explained) and B a mediating or intermediary block as far as it depends on A and is predictive with respect to C. As illustrated in Figure 1, in path 1, A corresponds to **Clinic**, C to **y**, and B to **Metabo**. Similarly, in path 2, A corresponds to **Metabo**, C to **y**, and B to **Clinic**.

The global effect of A on C corresponds to the amount of variance of C explained by A, while the partial effect of A on C, conditionally to B, is obtained by the amount of variance of C explained by A, taking into account the explanation of A and C by B. Let us denote $X_{A},\;X_{B}$ and $X_{C}$ as the data matrices associated with blocks A, B and C, respectively. Without loss of generality, we suppose that $X_{A},\;X_{B}$ and $X_{C}$ are column-wise centered. The Froebenius norm of a matrix is noted $\|.{\|}^{2}$.

The global effect of A on C is the explained variance accounted for regressing C on A (Figure 2a and Equation (1)). In Equation (1) of the regression model, $V_{AC}$ are the regression coefficients and $E_{AC}$ the residuals.

(1) $$X_{C}=X_{A}V_{AC}+E_{AC}$$

The global effect of A on C is therefore equal to

(2) $$\frac{\|X_{AC}V_{AC}{\|}^{2}}{\|X_{C}{\|}^{2}}$$

The determination of the partial effect of A on C, given B, requires first removing the linear dependence between B and C and between B and A, respectively (Figure 2b). The residuals of C on B, noted $E_{BC}$, and of A on B, noted $E_{BA},$ are thus retained:

(3) $$X_{C}=X_{B}V_{BC}+E_{BC}\;\mathrm{and}\;X_{A}=X_{B}V_{BA}+E_{BA}$$

The partial effect of A on C, given B, is determined as the explained variance accounted for regressing the residuals of C on the residuals of A thus obtained (Figure 2c):

(4) $$E_{BC}=E_{BA}V_{CBA}+E_{CBA}$$

The partial effect of A on C, given B, is therefore equal to

(5) $$\frac{\|E_{BA}V_{CBA}{\|}^{2}}{\|E_{BC}{\|}^{2}}$$

Finally, a repeated k-fold cross-validation procedure is performed to take into account sampling variability when estimating these effects. The “global” effect corresponds, therefore, to the average of the cross-validated percentages of explained variance of the output block by the input block. Similarly, the “partial” effect is estimated by averaging the cross-validated percentages of explained variance resulting from a regression between the residuals blocks.

#### 2.2.2. Determination of the Models by Means of PLS Regression

In a multidimensional framework, with data blocks gathering a large number of highly correlated variables, a PLS regression is carried out for each predictive model to prevent collinearity issues. Thus, the amount of global and partial explained variances are estimated from usual PLS regression models: PLS1 when only one variable is to be predicted, PLS2 otherwise. As far as the complexity of the model depends on the number of components, the optimal number of components to be retained in the different PLS models is tuned by repeated k-fold cross-validation associated with a stratified resampling and the application of the one standard error rule [8]. Such a rule leads to a good compromise between the parsimony and the quality of a model as it corresponds to the most parsimonious model having a cross-validated residual sum of squares lower than the smallest cross-validated sum of squares value plus one standard deviation.

Once the optimal model has been determined, the variable importance in the projection (VIP) values are evaluated in both cases, i.e., for models associated with global and partial effect assessments. Bootstrap mean and standard deviation of VIP indices were also computed. The threshold value of mean bootstrap VIP, to determine that a variable is important, was set independently for each path and each model based on the mean bootstrap VIP value diagrams. Finally, log2 fold-changes (Log2 FC) were calculated for each explanatory variable (on data neither mean-centered nor scaled to unit variance) to complete the model interpretation.

#### 2.2.3. Software and Implementation

Data analysis was performed under the R software (version 4.2.0, R Development Core Team, 2019), using ‘caret’ (createFolds() function) and ‘pls’ (plsr() function) R-packages. Both metabolomics and clinical variables were scaled to unit variance. The choice of the optimal numbers of components and the calculation of cross-validated explained variance was performed with 10-fold cross-validation repeated 50 times, with a resampling frame stratified on the **y** variable. Bootstrap mean and standard deviation of VIP indices were computed with 500 repetitions.

## 3. Results

### 3.1. Global and Partial Effect Estimations and Selected Variables by Means of VIP Indices

The explained variances and the number of components of each model are indicated in Table 1. For both paths, global and partial effect estimations were found to be similar. Moreover, the amount of explained variance associated with the partial effect showed greater variability than the global effect.

Concerning the global effect estimated for path 1, around 52% of the variance of **y** was explained by the **Clinic** block. Three clinical variables that are directly related to carbohydrate and lipid metabolism disturbances, in link with insulin resistance, namely “waist circumference”, “glycemia” and “triglyceridemia”, had variable importance in the projection (VIP) value higher than the threshold that was set to 1. Their observed and mean bootstrap VIP values and Log2 FC are provided in Table 2. These statistics for this global effect for all variables are provided in Supplementary Materials (Table S1).

For the partial effect estimated for path 1, after removing the amount of variance explained by the **Metabo** block, 23% of the variance of the **y** residuals was explained by the **Clinic** residuals. The two clinical variable residuals having important VIP values higher than 1 in this model, namely “waist circumference” and “systolic blood pressure”, are presented in Table 2. We observe that “waist circumference” was important both in the global effect and in the partial effect. All the observed and mean bootstrap VIP values for this partial effect are provided in Supplementary Materials (Table S1).

In path 2, the global effect of the **Metabo** block on **y** represented around 53% of the explained variance of **y**. The metabolites that were important in this global effect were found to be directly related to carbohydrate and lipid metabolism disturbances in link with insulin resistance. Nineteen annotated metabolomics variables had a significant mean bootstrap VIP value higher than the threshold that was set to 1.2 for this model (Table 3). These metabolites were previously identified as lipids (triglycerides, phosphatidylcholines, LDL, VLDL…), carbohydrates (hexoses, glucose), as well as amino acids (leucine, valine, glutamine) and derivatives [7]. All the Log2 FC (cases vs. controls) and all the observed and mean bootstrap metabolomics variable importance values in the projection in this global effect are provided in Table S2 in Supplementary Materials.

The partial effect estimated in path 2 of the **Metabo** block on **y**, i.e., after removing what was explained by the **Clinic** block, showed that around 22% of the variance of the **y** residuals was explained by the **Metabo** residuals. Sixteen residuals of metabolomics variables had a significant mean bootstrap VIP value higher than the threshold set to 1.2. Among them, the four previously identified are listed in Table 3. They were metabolites with endogenous and dietary origins (see Section 4) having different effects related to MetS, but not immediately linked to clinical parameters. Moreover, all the observed and mean bootstrap VIP values in this partial effect are indicated in Supplementary Materials (Table S2).

### 3.2. Comparison of Important Variables in the Global and Partial Effects

It is interesting to note that the most important variables in the global effect and those that become important in the partial effect were not the same in both paths, except the waist circumference that remained important in path 1 and PC(18:0_20:3), which is an alkylacyl phosphatidylcholine that remained important in path 2. In addition, the VIP indices of the variables in the partial effects had relatively high variability, which has already been noticed for the explained variance.

## 4. Discussion

### 4.1. Interest of Path Modeling Approaches

To the best of our knowledge, path modeling approaches have been applied with metabolomics to explain health-related outputs only in a few publications [9,10,11]. But no publications have already applied multiblock path modeling approaches with metabolomics for a clinical syndrome exploration. However, the path modeling or mediation approaches are of major interest compared to supervised multiblock methods, such as multiblock PLS regression, which search for components of the different data blocks providing the same or complementary information with respect to a block to be predicted without taking into account the links between the explanatory blocks.

Within the multidimensional framework of multiblock analysis, a path diagram is a convenient way to represent the conditional dependence relations between several blocks. Modeling these relationships may be achieved using components-based SEM (Structural Equation Modeling) methods such as PLS-PM [12], RGCCA [13] and GSCA [14]. More specifically, the approach we applied here, whose objective was to better understand a health-related predictive model, was inspired by an approach recently proposed under the name SO-PLS-PM, for Sequential and Orthogonalized Path Modeling PLS [15,16].

It is interesting to note that, in the particular case where the data blocks are restricted to a single variable, the path modeling approach refers to path analysis, on the basis of which so-called direct, indirect and total effects are defined [4,5].

### 4.2. Concepts of Global and Partial Effects

In order to clarify the difference between the concepts of direct, indirect and total effects from the global and partial effects used in this work, let us consider a unidimensional setting, where blocks A, B and C, shown in Figure 1, are restricted to single variables assumed to be standardized, denoted $x_{A}$, $x_{B}$ and $x_{C}$ for clarity.

The direct, indirect and total effects are assessed by combining the standardized regression coefficients (or path coefficients) of the multiple linear regression of $x_{C}$ as a function of $x_{A}$ and $x_{B}$, denoted $p_{x_{C},x_{A}}$ and $p_{x_{C},x_{B}}$ respectively, as well as the standardized regression coefficients of simple linear regression of $x_{B}$ as a function of $x_{A}$, which is nothing else than the linear correlation coefficient between $x_{A}$ and $x_{B}$, $r_{x_{A},x_{B}}$. The direct effect corresponds to $p_{x_{C},x_{A}}$, the indirect effect is obtained by the multiplication of $p_{x_{C},x_{B}}$ and $r_{x_{A},x_{B}}$, and the total effect is the sum of the direct and the indirect effects. It is equivalent to $r_{x_{A},x_{C}}$, the linear correlation coefficient between $x_{A}$ and $x_{C}$ [4,5]. Direct and indirect effects determined by means of the path coefficients are very popular because of specifically addressing causal analysis. Nevertheless, in a multidimensional framework, the path coefficients between A, B and C data matrices are no longer defined globally but have to be determined for each pair of individual variables involved in the three blocks, as in [9,17].

Our point of view was to consider instead, on the one hand, the linear correlation coefficient between $x_{A}$ and $x_{C}$, $r_{x_{A},x_{C}}$, which is the above defined total effect, and, on the other hand, the partial correlation coefficient between $x_{A}$ and $x_{C}$ conditionally of $x_{B}$, $r_{x_{A},x_{C}/x_{B}}$. The squared correlation and the squared partial correlation correspond to the explained variance accounted for the regression models between $x_{A}$ and $x_{C}$, on the one hand, and between $x_{A}$ and $x_{C}$ given $x_{B}$, on the other hand. They have been defined as the global effect (Equation (2)) and the partial effect (Equation (5)), respectively. In contrast to the use of direct and indirect effects, the evaluation of the explained variance, and hence the quantification of global and partial effects, are generalizable to the multidimensional case.

In our study, we could neither proceed to a selection of variables nor subdivide the explanatory blocks to make them unidimensional, notably because of the large amount of information in the metabolomic block and the risk of obtaining unidimensional blocks that would be uninterpretable. Therefore, to determine the global effect of A on C as well as its partial effect, we recommend using the explained variance of the corresponding regression models. It is worth noting that such an approach is the one proposed by Naes et al. within the framework of SO-PLS-PM [15,16], in which the way the indirect effect is determined is completely different from the classical approach adopted in path analysis. Consequently, we do not refer here to the direct and indirect effect terms, which may be confused with the terms used in the path analysis.

It is interesting to note that in our method, there is no weighting of the blocks. It could, therefore, be applied with limited risk of not highlighting important variables when explanatory data blocks are very different in terms of dimensions, information content with respect to a variable to be predicted, transformation or scaling.

### 4.3. Input for the Exploration of Metabolic Syndrome

The presented approach can significantly contribute to helping to interpret the links between clinical and metabolomics data, in particular for the exploration of clinical syndromes. Indeed, in such approaches, the strength of each link between the different datasets, considering the others, can be determined simultaneously. Additionally, as in multiblock analyses, the most important variables in these links can be used to highlight corresponding biological effects.

From a biological point of view, the present results highlighted the complementary information provided by metabolomics to clinical data and, reciprocally, in the MetS exploration. In particular, as expected, results showed that metabolomics is the measurement of metabolic phenotypes but also the reflection of the secondary functional deficits associated with MetS.

Presently, by a global effect, metabolomics data explained, as well as the **Clinic** dataset, the glycemic and lipid disturbances observed at the blood level in the case of MetS. When the partial effect is analyzed, i.e., when the information explained by the **Clinic** was removed from the metabolomics, metabolites further linked to dysfunctions were highlighted. These metabolites allowed a more systemic and comprehensive view of the processes involved in the syndrome.

It is first illustrated in path 1, where the most important clinical variables in the projection related to the global effects are measurements of blood biochemical parameters, whereas residuals of those important in the partial effect are, secondly, measures of adiposity and vascular dysfunction, respectively. Interestingly, waist circumference is important in both effects, as it could also be linked to the disturbance of the insulin action associated with the accumulation of abdominal fat. Indeed, over the last decades, adipose tissue has emerged not only as a key actor in multiple processes such as metabolism and adipogenesis but also as a very important endocrine organ, being able to secrete hormones and inflammation regulators [18]. Therefore, our results raise the question of waist circumference as a clinical measurement reflecting not only the absolute amount of intra-abdominal or visceral fat but also of subcutaneous adipose tissue, both having different and complex functions, which need to be further investigated within the emerging field of adipocyte biology.

Secondly, the importance of the residuals of some metabolomics variables (not explained by the clinical variables) in path 2 brought some statistical evidence of the independence of complex effects that support distinct physiological processes leading to MetS. In detail, PC(18:0_20:3) is an alkylacyl phosphatidylcholine both linked to lipid and cholesterol transports. It was associated with waist circumference, body mass index, C-peptide and leptin [19], but also with high blood pressure and dyslipidemia, which could explain its importance in both effects [20]. Secondly, 1,5-anhydroglucitol, recognized as a short-term marker of glycemic control, was recently identified as a circulating biomarker of the functional ß-cell mass of the islets of Langerhans, which produce insulin. In fact, a close association between 1,5-anhydroglucitol levels and poor glucose control was evidenced in type 2 diabetic patients, although not in nondiabetic subjects. It was shown that the loss of ß-cells was necessary and sufficient to decrease circulating 1,5-anhydroglucitol, not requiring hyperglycemia. It is, therefore, partially not immediately linked to glycemic disturbance [21,22,23,24]. Regarding betaine, this metabolite has both endogenous and exogenous origins, as it is a nutrient obtained from the diet (e.g., some green veggies, whole grains, and shellfish), but also synthesized de novo in the kidney and liver by choline oxidation [25,26]. It is an important osmoprotectant and methyl group donor with anti-inflammatory effects [27]. It has been shown that betaine was also inversely associated with serum non-HDL cholesterol, triglycerides, BMI, percent body fat, waist circumference, and systolic and diastolic blood pressure and positively associated with HDL cholesterol [28].

In the original publication [7], correlation analyses were used to explore the relationships between the molecular signature and clinical parameters. Their results highlighted the links between almost all significantly modulated metabolites and the five individual clinical criteria defining MetS, without that much specificity (i.e., a metabolite chemical family being related to several MetS criteria), revealing the interconnection of complex underlying metabolic processes and MetS components. The present approach allowed going further into the exploration of the relationships between MetS, its clinical criteria and its metabolic signature. Interestingly, the assessment of global and partial statistical effects reflecting orthogonal statistical links revealed corresponding physiopathological independent processes, which can be measured within single metabolomic variables.

## 5. Conclusions

In our study, a path-modeling method was implemented in a multidimensional context. This method can be easily applied with correlated variables and different blocks in terms of dimensions, transformations and normalizations. The interpretation of the results, based on the explained variances and VIP values, is also straightforward.

The determination of both global and partial effects, together with the identification of the most important variables from the associated models, highlighted the redundancy as well as the complementarity of the clinical and metabolomic information in the MetS explanation. In particular, the disturbances in lipid and carbohydrate metabolism, which exist in metabolic syndrome and are measurable at the plasma level, were highlighted by the important clinical or metabolomic variables in the global effects. Thus, these variables were often no longer important in the partial effect. In particular, in the partial effect of **Clinic** on **y**, given the presence of **Metabo** in the diagram, the residuals of functional variables became important. And in the partial effect of **Metabo** on **y**, given the presence of **Clinic** in the model, metabolic variables not explained by MetS clinical diagnostic variables were highlighted. The present developed approach is of major interest in deciphering the relationships between metabolomic data and clinical measurements, allowing us to go deeper into the interpretation of metabolomic data in the exploration of metabolic phenotypes of clinical syndromes.

## Figures and Tables

**Table 1:** Global and partial effects estimated for both paths described in Figure 1.

Effects	Explained Variance ± SD (%)	Number of PLS Components
**Path 1**		
**Clinic** => **y** (global effect)	52.37 ± 0.74	1
**Clinic** => **y**|**Metabo** (partial effect)	22.95 ± 1.85	2
**Path 2**		
**Metabo** => **y** (global effect)	53.43 ± 1.47	2
**Metabo** => **y**|**Clinic** (partial effect)	21.67 ± 3.83	2

**Table 2:** Identifications, VIP and Log2 FC of the most important variables in the effects of **Clinic** on **y** in path 1.

Important Variable in the Model	Identification	VIP	Mean Bootstrap VIP ± SD	Log2 FC ^1^ (Cases/ Controls)
**Global effect**				
WC	waist circumference	1.53	1.50 ± 0.11	0.23
GLY	glycemia	1.11	1.10 ± 0.13	0.36
TG	triglyceridemia	1.08	1.08 ± 0.13	0.86
**Partial effect**				
WC residual	waist circumference	1.44	1.41 ± 0.24	
SBP residual	systolic blood pressure	1.41	1.33 ± 0.24	

^1^ no Log2 FC was calculated for partial effects because of the relevance of Log2 FC on residual values.

**Table 3:** Identifications, VIP and Log2 FC of the most important metabolomics variables in the effects of **Metabo** on **y** in path 2.

Important Variable in the Model	Identification Reported in Comte et al. [7]	VIP	Mean Bootstrap VIP ± SD	Log2 FC ^1^ (Cases/Controls)
**Global effect**				
V5261	TG(16:0_18:1_18:1)	2.11	2.00 ± 0.26	0.06
V3854	PC(18:0_20:3)	1.94	1.79 ± 0.26	0.02
M179T471	Hexoses	1.88	1.77 ± 0.17	0.02
M101.0244T0.93	Hexoses	1.83	1.72 ± 0.17	0.02
BV_1.273_NMR	LDL, VLDL	1.82	1.76 ± 0.27	0.05
BV_5.23745012_NMR	D-α-Glucose	1.73	1.62 ± 0.17	0.01
V2975	PE(18:0_20:4)	1.66	1.59 ± 0.28	0.04
M261.1445T7.64	ɣ-Glutamyl-leucine	1.55	1.49 ± 0.23	0.03
M215.0328T0.91	Hexoses	1.52	1.41 ± 0.18	0.04
M203.0526T0.91	Hexoses	1.52	1.40 ± 0.23	0.02
M163.06T0.91_1	Hexoses	1.52	1.45 ± 0.24	0.04
M178T555	Glucosamine	1.44	1.30 ± 0.20	−0.04
M274T549	Glutamyl-glutamine	1.43	1.35 ± 0.30	0.06
M564.3308T14.67	LPC(18:2_0:0)	1.43	1.37 ± 0.24	−0.03
M146.0459T0.91	L-Glutamic acid	1.42	1.36 ± 0.28	−0.02
M520.3397T14.67	LPC(18:2_0:0)	1.36	1.31 ± 0.24	0.02
M223.0925T0.93	Hexahydroxyheptane hydrazide	1.35	1.30 ± 0.23	0.04
M118.0863T1.19	L-Valine	1.34	1.26 ± 0.21	0.01
**Partial effect**				
V3854 residual	PC(18:0_20:3)	2.21	1.71 ± 0.46	
M200T324 residual	1,5-anhydroglucitol	2.00	1.48 ± 0.45	
M118.0862T0.92 residual	Betaine	1.94	1.52 ± 0.56	
M174.0571T6.89 residual	2-(methoxyimino)- propanoic acid	1.26	1.24 ± 0.36	

^1^ no Log2 FC was calculated for partial effects because of the relevance of Log2 FC on residual values.

## Data Availability

The data supporting this manuscript are available in Supplementary data in Comte et al. (2021) [7].

## Supplementary Materials

The following supporting information can be downloaded at: https://www.mdpi.com/article/10.3390/metabo13030373/s1, Table S1: Identifications, Log2 FC, and VIP values of the clinical variables in path 1; Table S2: Identifications, Log2 FC, and VIP values of the metabolomics variables in path 2.
