# Supplementary material for: Global and Partial Effect Assessment in Metabolic Syndrome Explored by Metabolomics
Source: Metabolites. 2023 Mar 2;13(3):373. doi: 10.3390/metabo13030373 (PMC10058487; doi:10.3390/metabo13030373)
Supplement: Supplementary file 1 [file metabolites-13-00373-s001.zip › metabolites-2221461-supplementary.pdf]

SUPPLEMENTARY MATERIALS

Global and partial effect assessment in metabolic syndrome explored by metabolomics

Marion Brandolini-Bunlon[1,*], Benoit Jaillais[2], Véronique Cariou[2], Blandine Comte[1], Estelle Pujos-Guillot[1] and Evelyne Vigneau[2]

[1] Université Clermont Auvergne, INRAE, UNH, Plateforme d'Exploration du Métabolisme, MetaboHUB Clermont, 63000 Clermont-Ferrand, France

[2] Oniris, INRAE, StatSC, 44322 Nantes, France

* Correspondence: marion.brandolini-bunlon@inrae.fr

Supplementary Materials:

Table S1: Identifications, Log2 FC, and VIP values of the clinical variables in path 1.

Table S2: Identifications, Log2 FC, and VIP values of the metabolomics variables in path 2.

Table S1. Identifications, Log2 FC, and VIP values of the clinical variables in path 1.

Clinic variables	Identification	Log2 FC[1] (cases / controls)	Global effect (1 cp) Clinic => y			Partial effect (2 cp) Clinic residuals => y residuals		
			observed VIP	mean boot VIP	sd boot VIP	observed VIP	mean boot VIP	sd boot VIP
HDL	HDL cholesterol	-0.338	0.772	0.758	0.167	0.771	0.760	0.326
TG	triglyceridemia	0.861	1.081	1.076	0.134	0.620	0.624	0.209
WC	waist circumference	0.232	1.524	1.501	0.107	1.440	1.404	0.236
GLY	glycemia	0.359	1.114	1.097	0.127	0.362	0.381	0.194
DBP	diastolic blood pressure	0.059	0.342	0.354	0.192	0.904	0.891	0.237
SBP	systolic blood pressure	0.140	0.742	0.735	0.170	1.412	1.326	0.242

[1] log2 fold-changes calculated on the initial values.

Table S2. Identifications, Log2 FC, and VIP values of the metabolomics variables in path 2.

Metabo variables	Method	Identification	Log2 FC[1] (cases/ con- trols)	Global effect (1 cp) Metabo => y			Partial effect (2 cp) Metabo residuals => y residuals		
				Obser- ved VIP	mean boot VIP	sd boot VIP	Obser- ved VIP	mean boot VIP	sd boot VIP
M200 T324	HILIC neg	1,5-anhydroglucitol	-0.050	1.180	1.112	0.307	1.997	1.477	0.451
M187.0577 T1	C18 pos	1,5-anhydroglucitol	-0.053	1.224	1.181	0.288	1.158	0.895	0.402
M217.1071 T22.37	GCMS	1,5-anhydroglucitol	-0.051	1.021	1.004	0.281	0.857	0.926	0.344
M118.0862 T0.92	C18 pos	Betaine	-0.019	1.241	1.163	0.325	1.935	1.521	0.561
M368.3451 T42.66	GCMS	Cholesterol	-0.003	0.368	0.470	0.243	0.489	0.691	0.324
M361.1703 T23.75	GCMS	D-α-Glucose	-0.006	0.257	0.441	0.243	0.900	1.081	0.488
M435.1977 T23.75	GCMS	D-α-Glucose	0.002	0.284	0.444	0.239	0.865	1.099	0.451
M436.2037 T23.75	GCMS	D-α-Glucose	0.027	0.385	0.560	0.262	0.459	0.948	0.371
M62.0133 T23.76	GCMS	D-α-Glucose	0.114	0.813	0.835	0.271	0.753	0.950	0.318
BV_5.23745012	NMR	D-α-Glucose	0.009	1.727	1.616	0.174	0.773	0.636	0.301
M319.1593 T24.13	GCMS	D-β-Glucose	0.012	0.534	0.657	0.264	0.477	1.026	0.440
M261.1445 T7.64	C18 pos	ɣ-Glutamyl-leucine	0.030	1.544	1.488	0.231	0.712	0.624	0.344
M274 T549	HILIC neg	Glutamyl-glutamine	-0.028	1.432	1.349	0.295	1.281	1.010	0.444
V7346	LIPIDO	GM3(42:2)	-0.025	0.907	0.869	0.242	0.817	0.622	0.337
M188 T139	HILIC neg	Indole-3-propionic acid	-0.053	1.143	1.102	0.279	0.966	0.791	0.404
M132.1019 T1.2_2	C18 pos	Isoleucine	0.014	1.172	1.141	0.255	0.390	0.493	0.309
M86.0963 T1.21	C18 pos	Isoleucine	0.010	1.063	1.044	0.274	0.255	0.414	0.269
M117.0748 T7.08_2	GCMS	Lactic acid	-0.001	0.159	0.450	0.250	0.372	1.003	0.417
M146.0459 T0.91	C18 neg	L-Glutamic acid	0.063	1.415	1.359	0.282	0.749	0.676	0.402
M496.3397 T15.17	C18 pos	LPC(16:0_0:0)	-0.006	0.859	0.809	0.229	0.544	0.496	0.269
M540.3308 T15.17	C18 neg	LPC(16:0_0:0)	-0.008	0.973	0.917	0.220	0.406	0.448	0.255
V839	LIPIDO	LPC(P-16:0_0:0)	-0.005	0.760	0.644	0.194	1.125	0.792	0.425
M522.3554 T15.56	C18 pos	LPC(18:1_0:0)	-0.015	1.029	0.978	0.230	0.645	0.504	0.305
M520.3397 T14.67	C18 pos	LPC(18:2_0:0)	-0.018	1.355	1.308	0.240	0.494	0.540	0.320
M564.3308 T14.67	C18 neg	LPC(18:2_0:0)	-0.037	1.426	1.371	0.242	0.618	0.608	0.363
V1115	LIPIDO	LPC(20:1_0:0)	-0.018	1.109	1.023	0.211	0.397	0.462	0.278
M116.0706 T0.98	C18 pos	L-Proline	0.009	0.678	0.698	0.298	0.641	0.617	0.342
M118.0863 T1.19	C18 pos	L-Valine	0.009	1.337	1.263	0.211	0.288	0.504	0.274
V3854	LIPIDO	PC(18:0_20:3)	0.020	1.937	1.784	0.255	2.205	1.713	0.460

V3734	LIPIDO	PC(P-39:5)	-0.021	0.574	0.602	0.284	0.761	0.650	0.321
V2975	LIPIDO	PE(18:0_20:4)	0.038	1.663	1.590	0.283	1.169	0.918	0.408
M130.0862 T1.19	C18 pos	Pipecolic acid	-0.031	1.068	1.026	0.320	1.072	0.869	0.441
M144.1018 T1.04	C18 pos	Proline Betaine	0.051	0.472	0.538	0.273	0.470	0.599	0.339
V5261	LIPIDO	TG(16:0_18:1_18:1)	0.062	2.113	1.999	0.261	1.144	0.962	0.398
M61.0393 T0.95	C18 pos	Urea	0.009	0.697	0.729	0.237	1.014	0.819	0.438
M174.0571 T6.89	GCMS	2-(methoxyimino)-propanoic acid	0.044	0.866	0.906	0.237	1.259	1.244	0.362
M178 T555	HILIC neg	Glucosamine	0.022	1.439	1.302	0.199	0.695	0.648	0.385
BV_1.273	NMR	LDL, VLDL	0.018	1.818	1.763	0.268	0.353	0.458	0.277
M292.1333 T15.77	GCMS	Threonic acid	-0.034	0.517	0.597	0.255	0.740	0.959	0.290
M261.0113 T0.92	C18 pos	Caffeic acid 3 or 4-sulfate	0.008	1.238	1.175	0.227	0.639	0.608	0.301
M223.0925 T0.93	C18 pos	Hexahydroxyheptane hydrazide	0.040	1.350	1.299	0.231	0.871	0.706	0.362
M163.06 T0.91_1	C18 pos	Hexoses	0.040	1.519	1.446	0.239	0.504	0.521	0.303
M101.0244 T0.93	C18 neg	Hexoses	0.046	1.825	1.724	0.171	0.478	0.455	0.237
M203.0526 T0.91	C18 pos	Hexoses	0.014	1.521	1.403	0.234	0.595	0.484	0.237
M215.0328 T0.91	C18 neg	Hexoses	0.018	1.523	1.409	0.179	0.590	0.496	0.310
M179 T471	HILIC neg	Hexoses	0.021	1.876	1.771	0.171	0.512	0.435	0.221
M504.3085 T15.12	C18 pos	LPC	0.009	0.820	0.736	0.288	1.184	1.030	0.464
M114.0661 T0.95	C18 pos	Unknown	0.004	0.503	0.541	0.215	0.939	0.710	0.384
M117.0726 T6.09	GCMS	Unknown	-0.003	0.136	0.406	0.204	0.648	0.838	0.321
M165.0697 T8.42	GCMS	Unknown	0.004	0.218	0.413	0.212	0.461	0.594	0.352
M174.1093 T23.8	GCMS	Unknown	0.010	0.453	0.561	0.282	0.353	0.957	0.380
M191.0913 T8.63	GCMS	Unknown	-0.014	0.415	0.540	0.240	0.411	0.801	0.321
M205.1067 T10.37	GCMS	Unknown	-0.008	0.105	0.393	0.211	0.575	0.563	0.291
M218 T213	HILIC neg	Unknown	0.027	1.156	1.026	0.349	1.697	1.310	0.564
M246.1335 T16.84	GCMS	Unknown	0.036	0.592	0.682	0.257	0.147	0.881	0.343
M267.0853 T14.44	GCMS	Unknown	0.050	0.278	0.412	0.259	0.929	0.832	0.479
M299.0723 T10.38	GCMS	Unknown	0.006	0.352	0.497	0.235	1.291	1.107	0.357
M313.256 T26.23	GCMS	Unknown	0.010	0.660	0.626	0.280	1.252	1.011	0.432
M318.1493 T27.96	GCMS	Unknown	-0.000	0.150	0.465	0.232	0.681	1.014	0.420
M72.08 T24.9	GCMS	Unknown	-0.042	0.864	0.886	0.252	0.838	1.054	0.306
M218.1024 T13.73	GCMS	Unknown	0.003	0.541	0.573	0.252	0.856	1.098	0.334
M72.0354 T9.76	GCMS	Unknown	-0.002	0.179	0.431	0.217	0.333	0.902	0.404
V1738	LIPIDO	Unknown	-0.003	0.610	0.576	0.303	1.592	1.219	0.549
V2165	LIPIDO	Unknown	0.006	1.069	0.895	0.251	2.118	1.567	0.427
V4198	LIPIDO	Unknown	0.023	1.065	0.922	0.293	1.722	1.296	0.422
V5931	LIPIDO	Unknown	0.015	1.021	0.876	0.288	2.253	1.636	0.522

V983	LIPIDO	Unknown	0.005	1.227	0.938	0.293	1.727	1.255	0.438
M102.041 T7.99	GCMS	Unknown	-0.024	0.546	0.588	0.224	0.856	0.952	0.337
M104.1073 T0.88	C18 pos	Unknown	0.003	0.328	0.447	0.219	0.727	0.617	0.311
M116.0892 T7.74	GCMS	Unknown	0.003	0.187	0.435	0.223	0.090	0.926	0.387
M131.0885 T8.14	GCMS	Unknown	0.030	0.668	0.747	0.259	0.840	1.106	0.338
M146 T556	HILIC neg	Unknown	0.049	0.478	0.558	0.248	0.894	0.731	0.379
M159 T578	HILIC neg	Unknown	-0.047	1.095	0.934	0.401	2.321	1.831	0.553
M172.0652 T9.13	GCMS	Unknown	-0.062	0.363	0.494	0.235	0.244	0.692	0.287
M177.0754 T8.64	GCMS	Unknown	0.036	0.785	0.852	0.238	1.322	1.388	0.338
M178 T285	HILIC neg	Unknown	-0.022	1.245	1.193	0.289	1.209	0.934	0.460
M185.1096 T15.77	GCMS	Unknown	-0.028	0.760	0.737	0.237	1.544	1.461	0.347
M185 T80	HILIC neg	Unknown	-0.022	1.135	1.023	0.348	1.643	1.258	0.545
M189.0924 T9.75	GCMS	Unknown	-0.000	0.089	0.391	0.243	0.148	0.897	0.466
M190.0898 T9.76_2	GCMS	Unknown	-0.000	0.140	0.436	0.244	0.503	0.988	0.471
M204.0992 T25.67	GCMS	Unknown	-0.004	0.049	0.368	0.206	0.522	0.604	0.356
M204.099 T24.58	GCMS	Unknown	-0.046	0.523	0.611	0.263	0.708	0.839	0.394
M225 T472	HILIC neg	Unknown	0.028	1.141	1.077	0.219	0.310	0.456	0.267
M268 T461	HILIC neg	Unknown	0.047	1.328	1.254	0.222	0.379	0.480	0.269
M273.0969 T21.66	GCMS	Unknown	-0.020	0.448	0.607	0.218	0.276	0.989	0.391
M274.9885 T0.91	C18 neg	Unknown	0.041	1.476	1.407	0.199	0.335	0.426	0.246
M293 T579	HILIC neg	Unknown	0.035	1.287	1.218	0.193	0.843	0.778	0.409
M305.918 T0.9	C18 neg	Unknown	0.040	1.175	1.131	0.205	0.442	0.521	0.329
M333.1365 T24.62	GCMS	Unknown	0.053	0.864	0.842	0.259	1.870	1.568	0.404
M357.1124 T20.38	GCMS	Unknown	-0.035	0.622	0.698	0.269	0.373	0.902	0.357
M456.1862 T28.17	GCMS	Unknown	-0.010	0.870	0.758	0.279	1.591	1.406	0.428
M55.0175 T0.92	C18 pos	Unknown	0.027	1.231	1.183	0.279	0.581	0.557	0.314
M618 T458_1	HILIC neg	Unknown	-0.026	0.655	0.685	0.270	0.715	0.699	0.408
M698.7868 T9.52_1	C18 pos	Unknown	0.011	0.593	0.601	0.280	0.615	0.650	0.349
M698 T452	HILIC neg	Unknown	0.015	0.604	0.589	0.264	1.342	1.097	0.469
M733.3319 T8.51	C18 pos	Unknown	0.002	0.463	0.473	0.217	0.844	0.778	0.429
M74.0392 T23.43	GCMS	Unknown	0.000	0.470	0.493	0.268	0.687	0.739	0.366
M76.0288 T9.76	GCMS	Unknown	0.013	0.427	0.496	0.226	0.659	0.704	0.354
M87 T265	HILIC neg	Unknown	0.037	1.143	1.023	0.312	1.544	1.194	0.429

M95 T977	HILIC neg	Unknown	-0.025	0.319	0.507	0.255	0.535	0.552	0.348
V4330	LIPIDO	Unknown	-0.027	1.061	1.023	0.244	0.621	0.570	0.291
V5157	LIPIDO	Unknown	-0.017	0.805	0.715	0.244	1.180	0.871	0.438

[1] log2 fold-changes calculated on the initial values.
